# Drinking Water Quality Surveillance in a Vulnerable Urban Ward of Ahmedabad

**DOI:** 10.4236/health.2014.611143

**Published:** 2014-05-01

**Authors:** Veena Iyer, Nandini Choudhury, Gulrez Shah Azhar, Bhushan Somvanshi

**Affiliations:** Indian Institute of Public Health Gandhinagar, Sardar Patel Institute for Economic and Social Research Campus, Thaltej, India

**Keywords:** Ahmedabad, Gujarat, Drinking Water Quality, Water Quality Surveillance, Urban India, WHO Drinking Water Quality

## Abstract

The World Bank estimates that 21% of all communicable diseases in India are related to unsafe water with diarrhoea alone causing more than 0.1 million deaths annually. The WHO drinking water surveillance parameters of quality, quantity, accessibility, affordability and continuity were assessed in one vulnerable ward of Ahmedabad—a fast growing city in Western India. Interviews with key informants of the ward office, health centre and water supply department, secondary analysis and mapping of field test reports and a questionnaire-based survey of different household types were conducted. We found that Ahmedabad Municipal Corporation (AMC) supplies water to the ward intermittently for two hours during the day. Housing society clusters supplement their AMC water supply with untested bore-well water. The water quality surveillance system is designed for a twenty-four-hour piped distribution of treated surface water. However, in order to maintain surveillance over an intermittent supply that includes ground water, the sampling process should include periodic surveys of water actually consumed by the citizens. The laboratory capacity of the Central Water Testing Laboratory should expand to include more refined tests for microbial and chemical contamination.

## 1. Introduction

Water is essential for sustaining all life forms and access to clean and safe drinking water is a basic human need. Latest estimates of the Joint Monitoring Programme for Water Supply and Sanitation state that the world is on track to meet the Millennium Development Goals target for drinking water of “halving by 2015 the proportion of people without sustainable access to safe water and basic sanitation” [1] [2]. But this progress has been uneven. An estimated 768 million people did not use an improved source for drinking-water in 2011 and 185 million still depended on surface water (from lakes, rivers, dams, or unprotected dug wells or springs) for their daily drinking-water needs [3] [4].

India has only 3% of the world’s fresh water with 20% of its population. Although, 92% - 96% of the urban population could access improved sources of water by 2010, 4% - 8% continues to use unimproved sources [4]-[6]. The World Bank estimates that, in spite of the improvement in provision for drinking water, 21% of all communicable diseases in India are related to unsafe water [7], with diarrhoea alone causing more than one hundred thousand deaths annually [8].

Groundwater constitutes 85% of the source of drinking water in India [9] [10] and none of the major Indian cities have a continuous water supply [11]. The Ministry of Urban Development, Government of India commissioned a study in 1999 to assess the status of water supply, sanitation and solid waste management in 305 selected Metropolitan, Class I and Class II cities and towns (of population more than 100,000). This study found that the coverage by the formal water supply was 94%. Paradoxically, coverage did not ensure quantity, quality, duration of supply or the mode of provision. In spite of 100 percent coverage in twenty-two sampled urban centres, regular daily supply was not ensured due to acute water shortage. Six sampled urban centres did not have individual house service connections. In most of the sampled cities, the duration of water supply ranged between 1 and 6 hours daily. The study also found that 43% of the sampled urban centres depended entirely on surface water, 34% depended entirely on groundwater while 22% used both surface and groundwater sources [12] [13].

The city of Ahmedabad located on the banks of a seasonal river—Sabarmati, has begun to receive water from the Narmada canal of the Sardar Sarovar project since the year 2000. This now supplements the underground water from French wells and Bore wells. This development has slowed the groundwater table depletion, but groundwater extraction is still widespread, especially in areas not provided by municipal water supply [14].

We investigated the drinking water surveillance parameters in one vulnerable ward of Ahmedabad. The WHO defines drinking water surveillance as the “the continuous and vigilant public health assessment and review of the safety and acceptability of drinking-water supplies”. It must include an assessment of quality, quantity, accessibility, affordability and continuity of the drinking-water supply [15].

## 2. Methods

Our selection of this study ward was based on the municipal health officials’ categorisation of this particular ward as the most vulnerable in the city. It is situated within the old city limits with more than 85% of its population residing in slums.

The data collection for the study took place in the summer of 2012. A questionnaire was developed based on the interviews we conducted with the municipal water supply officials. This was pilot tested in the neighbouring areas before being administered at the study site. The researchers made several visits to the study ward to understand the distribution of the household clusters and water supply across the ward.

We used a mixed methodology of:

### 2.1. Interviews with Key Informants

We interviewed field level sanitary and health workers and supervisors posted at the ward office and health centre. We also interviewed water supply personnel (engineers and operators) at the main water storage and distribution facility located in our study ward and personnel at the Water Testing Laboratory.

### 2.2. Secondary Analysis and Mapping of Field Test Reports

Field testing for water samples by the sanitary inspector is recorded on a standard format in a register including the date, source of water supply, site of sample collection and the result. We obtained this list of locations and results of water sample field tests in the study ward for a six month period from October 2011 to March 2012. These were analysed and mapped using ArcGIS software.

### 2.3. A Short Survey

**Figure 1:**
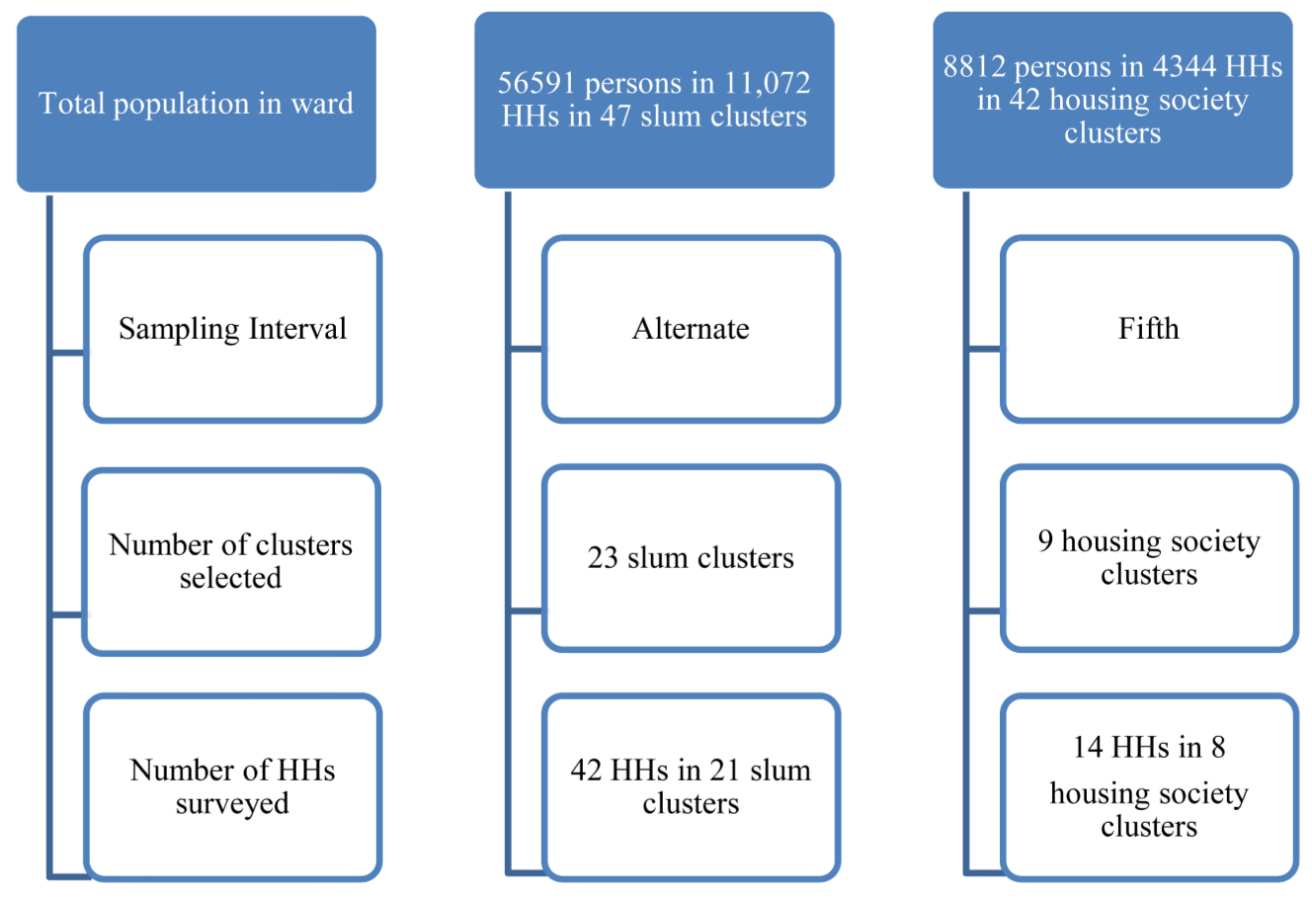
Description of survey methodology.

We obtained lists of population settlements from field health supervisor and used this to survey households in different types of tenements across the ward. The list divided the ward population of 65,403 into two groups; 86% of them living in 11,072 households (HHs) grouped into 47 slum clusters and 14% living in 4344 households (HHs) grouped into 42 housing society clusters. We sorted these cluster types separately in ascending order of population sizes. We sampled every alternate slum cluster from 47 slum clusters and every fifth housing society from 42 housing society clusters. (Slum clusters were oversampled because we were more interested in the poorer population of the ward.) We surveyed two HHs from every sampled cluster, one close to and one away from the municipal water line on the main road which supplies each cluster. This approach enabled us to assess the reach of the municipal water to the entire slum or society cluster and check for any disparities in supply between the proximal and distal households. The survey questionnaire was administered to a member of the household, the female head being the first preference. We finally surveyed 42 HHs out of 11,072 HHs in 21 slum clusters and 14 HHs from 4344 HHs in 8 housing society clusters. In two societies we sampled only one HH each because all the apartments were grouped around the water connection. Therefore the total tally was 56 households from 29 locations (Figure 1). The data was analysed in MS-Excel and EpiInfo7.

The data was stored securely on a hard disk and no personally identifiable information was collected. All participants were assured of confidentiality of their responses and a formal verbal consent was taken before starting the interview. Ethical clearance for the study was sought from the Institutional Ethics Committee of the Public Health Foundation of India. Support letters were also arranged from the Ahmedabad Municipal Corporation.

## 3. Results and Discussion

Our interviews revealed that Ahmedabad city receives approximately 1 billion litres of water per day, which averages out to nearly 180 litres per head. Supply is maintained for 2 hours every morning throughout the year (6 to 8 am) and during summers, for an additional one hour in the evenings. Water supply is not metered anywhere in Ahmedabad and therefore the actual quantity of water consumed by residents is not recorded.

The Central Water Testing laboratory tests at least 200 to 250 routine water samples (3 to 4 from each of 64 wards) each day. Additional samples are processed based on public complaints and during focal outbreaks of waterborne diseases. These samples are subjected to the following tests—turbidity, alkalinity, hardness, ${\mathrm{Ca}}^{2+},{\mathrm{Mg}}^{2+},{\mathrm{Cl}}^{-},{\mathrm{SO}}_{4}^{2-}$, total dissolved solids, before being tested for faecal indicators (total coliform and fecal *E. coli*).

The Sanitary Inspector of every ward sends two water samples to the Central Water Testing laboratory and carries out 10 Orthotoludene field tests for residual chlorine from across the ward each day. The water samples for the field tests as well as the laboratory tests are collected during morning supply hours. There was no map of the existing water supply network and water sampling was not related to the physical organisation of the distribution system. Sampling is based on an arbitrary pattern of collecting samples from select slum clusters, municipal schools, public offices, etc. which has been set in practice over the years and on complaints from residents.

**Figure 2:**
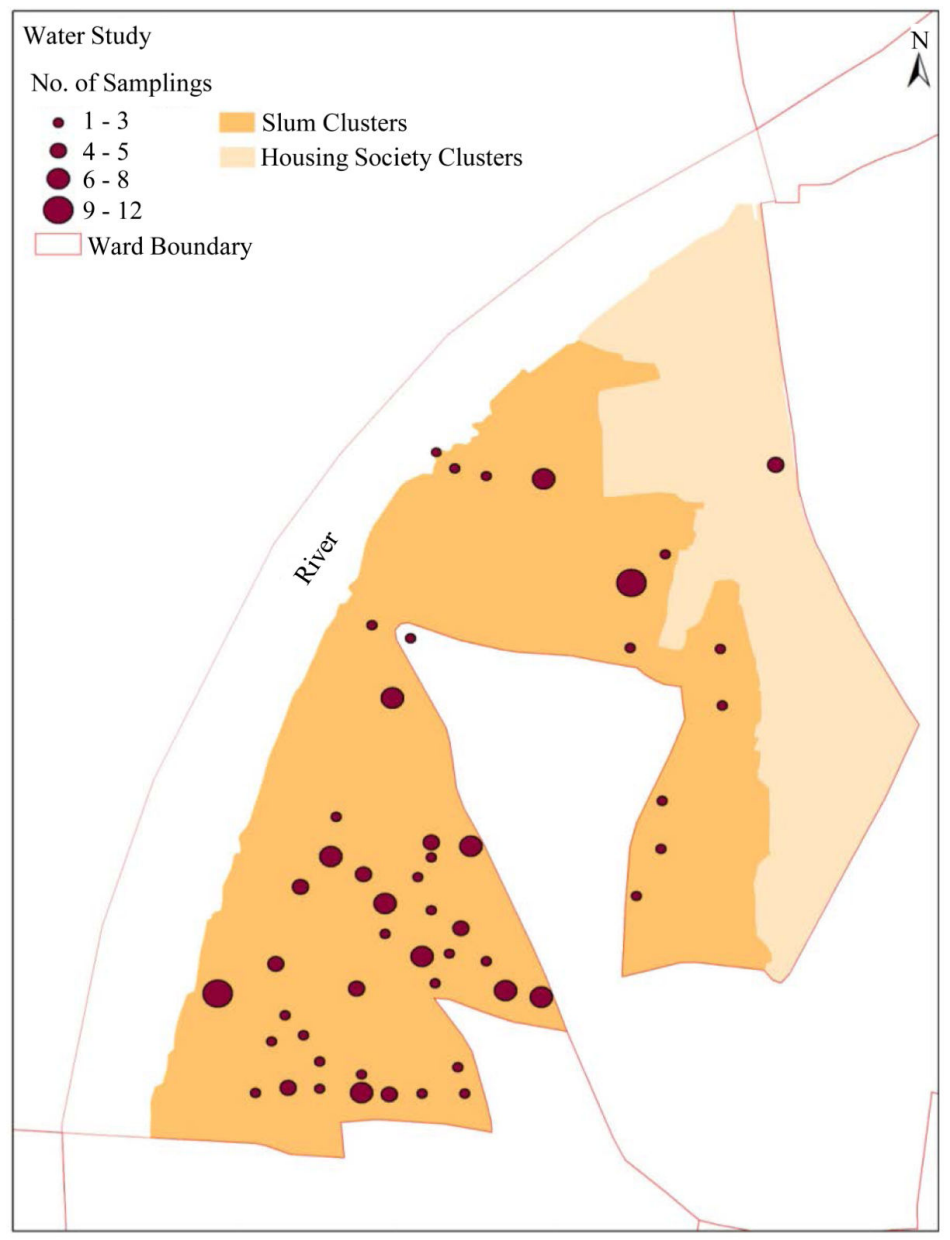
Map showing water sampling areas in the study ward.

Analysis and mapping of the records of 217 field tested water samples in the study ward over a six month period from October 2011 to March 2012 showed that 1) all samples had been reported fit; 2) most slum clusters were sampled repeatedly; 3) but none of the housing society clusters were covered by the sampling process (Figure 2).

The water supply department personnel of the ward examined the list of population settlements we had obtained from field health supervisor and marked out the most probable source of water (municipal supply, bore well or both) and the probable purification method used in each of the settlements. They estimated that of the 42 housing society clusters, only two supplement their municipal supply with bore-well water and one completely depends on bore well water for all of their water needs, and 12 of the 42 housing society clusters could afford small home-based Reverse Osmosis systems. Our survey showed this to be an underestimate.

### 3.1. Accessibility and Continuity

Our site visit revealed that, except for a tiny cluster of 250 HHs, 3.2% of the ward population which drew water from common stand posts, municipal water supply reached into the smallest and poorest of homes. Our survey of half of all the slum clusters found that all slum households depended on municipal water supply. Of the 8 housing society clusters, two tapped into groundwater while the rest depended on municipal supply.

### 3.2. Quantity

A visual estimation of the volume of stored water showed all HHs stored an average of 35 to 50 litres of drinking water and 600 to 1300 litres for other purposes (Table 1). Of the 42 slum HHs, 16% (7 HHs) used either mechanical or motorised pumps during water supply hours to get additional water. Two of the 8 housing society clusters used motorised pumps to draw ground water into their storage tanks.

The proportion of HHs that were dissatisfied with the quantity and/or pressure of water during summer was 45% in the slums (19 out of 42 HHs), and 50% (7 out of 14 HHs) in the society clusters. This dissatisfaction reduced to 26% in the slums HHs (11 out of 42 HHs) and 21% (3 out of 14 HHs) society HHs during the rest of the year (Table 1).

### 3.3. Quality

All slum and 80% of housing society households directly collected municipal water for drinking after simple sieving. The rest used Reverse Osmosis systems to purify water stored in overhead tanks for drinking.

This city’s supply of 1 billion litres of water per day averages to 180 litres per head. This falls within the range of 100 to 200 litres per capita, estimated by WHO as the amount likely to be collected when there is optimal access to water. At this level of water usage, public health risk due to poor hygiene is expected to be very low [15]. Our survey data bears out that the WHO water standards were met in the study ward.

The water supply in Ahmedabad is unmetered and water tax is included in annual property tax bills. Thus, water is affordable in the city, the bottleneck being the restricted supply hours and poor pressure of water. Citizens, depending on their affluence level, circumvent these shortcomings using one or more methods; by storing large quantities of water, by applying hand or motorized pumps directly on the municipal water line or by tapping into groundwater sources.

In our study ward, we found that poorer ward residents depended completely on the municipal water for both quality and quantity on a day to day basis. Each day, they separately collect and store water for drinking and for other purposes. Having larger families, and unable to afford storage facilities, they spent considerable time collecting and storing water.

The richer households are able to ensure quality and quantity of water they require by investing in permanent storage solutions like cement or plastic tanks. They have the option of either collecting the direct municipal supply water or use various types of water purifiers for drinking. Interestingly, in these richer housing societies, filtered and chlorinated municipal water is collected in underground storage tanks. Here, it is mixed with groundwater and thus rendered non-potable. This mixture is pumped to overhead tanks, and some of this now non-potable water is processed through Reverse Osmosis systems or other commercial water purifiers in individual households, for drinking.

The engineers responsible for water supply of the ward were unaware and unable to estimate how much piped water is consumed in the study ward and the extent of groundwater usage. They surmised that since this ward was upstream, there wouldn’t be any water shortage in this area.

The groundwater of Ahmedabad is known to have high levels of iron, fluoride, chloride and nitrates ([16] [17]). The Municipal Corporation as well as richer housing societies routinely resort to supplementing their water supply with ground water. The present drinking water surveillance system of the municipal corporation is designed for a twenty-four hour piped distribution of treated surface water. In reality, the water supply is neither 24 hours nor exclusively treated surface water. Therefore, as suggested by WHO [15], the water sampling process in the city needs to include periodic surveys of water actually consumed by the citizens. The Central Water Testing Laboratory needs to expand its laboratory capacity to include more refined tests for microbial and chemical contamination.

The WHO drinking water quality guidelines recommend that for every 50,000 population in a city with more than five million populations, faecal indicator testing in distribution systems should be carried out on a minimum of 12 samples with an additional 600 samples every year. By these standards, if the entire city had had an interconnected water supply network with positive pressure in the system 24 hours of the day, the city would require to test 2040 samples every year [15]. However, the Central Water Testing Laboratory tests a minimum of 224 water samples for faecal indicators every day and 67,200 samples every year. The water samples are subjected to only basic physical and chemical analysis. Capacity to test heavy metals (lead, cadmium, mercury, arsenic, and nitrates), trihalothanes and fluorides and advanced microbial tests for bacteriophages and spores are presently not available.

### 3.4. Strengths and Weakness of Our Study

We studied only a single ward of the city, therefore, the generalizability of these findings for the city are limited. Also the ward is located close to the water supply source for the city so the researchers suspect a better water availability in the study ward than the rest of the city. The investigators did not look for the existence of water vendors and thus cannot comment on purchase of water (and its financial implications) in times of non-supply of municipal water.

## 4. Conclusion

In conclusion, the water sampling strategy for the city of Ahmedabad needs to include periodic surveys of water actually consumed by the community and the testing strategy needs to incorporate tests for viruses, heavy metals, fluorides, trihalothanes and surface contaminants at appropriate intervals of time and distance in piped water, using maps of the water distribution systems. There is a need for consultations with experts to adjust the numbers and sophistication of tests to appropriately reflect available scientific knowledge about Drinking Water Quality Surveillance. Also, a practice of Sanitation Risk Scoring by Sanitary Inspectors of the city needs to be developed and implemented. Although there are official references to reducing groundwater exploitation [17], guidelines need to be developed for tapping into underground aquifers in the country.

## Figures and Tables

**Table 1:** Salient findings from the households’ survey of the ward.

	Slum clusters	Society clusters
No. of slums/societies	21	8 (+1 refused)
No. of HHs	42	14
Persons per HH	6	5
**Source of drinking water**	Municipal supply	6 municipal supply and 2 supplemented with private borewell
**Quantity**		
Water stored (lt/HH)		
Drinking	41	28
Cleaning	822	1320
Per capita presence of drinking water (litres)	7	6
Per capita presence of other water (litres)	143	286
HHs complaining of low pressure and/or less quantity supply in summer rest of the year	19 (45%) 11 (26%)	7 (50%) 3 (15%)
HHs using pumps	7 (16.67%)	2 (14.3%)
**Drinking water practices**		
Municipal water used directly for drinking	42 (100%)	11 (75% )
Earthenware pots used for storing drinking water	42 (100%)	13 (92.9%)
